# Long-Term Effects of the Cleaner Fish *Labroides dimidiatus* on Coral Reef Fish Communities

**DOI:** 10.1371/journal.pone.0021201

**Published:** 2011-06-24

**Authors:** Peter A. Waldie, Simon P. Blomberg, Karen L. Cheney, Anne W. Goldizen, Alexandra S. Grutter

**Affiliations:** The School of Biological Sciences, The University of Queensland, St Lucia, Queensland, Australia; Nanyang Technological University, Singapore

## Abstract

Cleaning behaviour is deemed a mutualism, however the benefit of cleaning interactions to client individuals is unknown. Furthermore, mechanisms that may shift fish community structure in the presence of cleaning organisms are unclear. Here we show that on patch reefs (61–285 m^2^) which had all cleaner wrasse *Labroides dimidiatus* (Labridae) experimentally removed (1–5 adults reef^−1^) and which were then maintained cleaner-fish free over 8.5 years, individuals of two site-attached (resident) client damselfishes (Pomacentridae) were smaller compared to those on control reefs. Furthermore, resident fishes were 37% less abundant and 23% less species rich per reef, compared to control reefs. Such changes in site-attached fish may reflect lower fish growth rates and/or survivorship. Additionally, juveniles of visitors (fish likely to move between reefs) were 65% less abundant on removal reefs suggesting cleaners may also affect recruitment. This may, in part, explain the 23% lower abundance and 33% lower species richness of visitor fishes, and 66% lower abundance of visitor herbivores (Acanthuridae) on removal reefs that we also observed. This is the first study to demonstrate a benefit of cleaning behaviour to client individuals, in the form of increased size, and to elucidate potential mechanisms leading to community-wide effects on the fish population. Many of the fish groups affected may also indirectly affect other reef organisms, thus further impacting the reef community. The large-scale effect of the presence of the relatively small and uncommon fish, *Labroides dimidiadus,* on other fishes is unparalleled on coral reefs.

## Introduction

On coral reefs, cleaning organisms - which include shrimps and fishes - perform the function of removing ectoparasites from ‘client’ organisms, usually reef fishes [1]. Cleaning behaviour has been used as a classic example of mutualism and, recently, to test cooperation theory [2]. Surprisingly, the health benefit to clients, in terms of body size, has never been measured [3] nor have any mechanisms involved in effects on fish communities [4], [5] been elucidated. On Atlantic and Indo-Pacific coral reefs, cleaner fishes interact with many client fish species [5]–[7]. The most common Indo-Pacific cleaner fish, *Labroides dimidiatus* [8], inspects an average 2297 clients day^−1^ and consumes an average 1218 ectoparasites day^−1^ [9]. Individual clients are often cleaned repeatedly, some up to 144 times day^−1^ [10]. Cleaner fishes often reside in ‘cleaning stations’ [3]; this site fidelity makes them an ideal model system for the study of localised effects of cleaning interactions.

There has been considerable debate about the mutualistic nature of cleaning symbioses. Benefits to cleaners are well documented; cleaners enjoy nutritional rewards from eating ectoparasites and protection from predation [3]. The benefit of cleaning to clients, however, remains contentious. Fish parasites can lower host growth, recruitment, and fecundity, and increase mortality [11], [12]. They have also been shown to affect fish foraging, swimming, and anti-predator behavior [13]. Thus, variation in parasite loads can lead to changes in their host community. However, early experimental removals of cleaner fish found no effects on ectoparasite or fish numbers after the removal of *L. phthirophagus* for one and seven months and *L. dimidiatus* for six months and two years [14]–[18].

In contrast, the removal of *L. dimidiatus* affected clients in three experiments. A short-term study (24 h and 12 d) at Lizard Island found that caged *Hemigymnus melapterus* had more and different sizes of parasitic isopods in the absence of cleaners, compared with controls [19], [20]. After 4–20 months, in the Red Sea, the species richness of ‘visitor’ (fish species that can move between patch reefs) and ‘resident’ (site-attached fish species) clients were reduced; however, fish abundance was not measured [4]. After 18 months, at Lizard Island, the species richness and abundance of visitors were reduced; however, no effect on resident species richness was detected [5]. A reduction in visitors could simply involve a change in visitation rates to reefs; in residents, the presumption is that it is more likely due to lower survivorship or recruitment [5]. Whether cleaner fish affect resident abundance over the long term (>6 months) or affect juveniles, however, has never been examined. Most importantly, the effect of cleaning on client fish fitness, including fish size, a common measure of condition and growth in fishes [21], has never been measured.

We investigated the long-term effects of cleaners on fish communities at Lizard Island in the longest study of its kind. We used an ongoing study in which patch reefs at two sites had been kept free of *L. dimidiatus* for 8.5 years, while similar control reefs had not had cleaner fish removed. First, to determine whether cleaning affects client size, we surveyed two common resident client fishes, *Pomacentrus moluccensis* and *P. amboinensis,* whose life spans are around eight (Shalan-Louise Bray, unpub. data) and six years (Mark I. McCormick, unpub. data.), respectively. Therefore, many individuals had experienced these experimental conditions for their entire lives. For each of these two species on each reef, we measured the sizes of all individuals and their total abundance. Second, we recorded the abundance and species richness of residents, juvenile visitors, and adult visitors on experimental removal and on control reefs.

## Results

### Size distribution and abundance of two resident damselfish species

**Figure 1:**
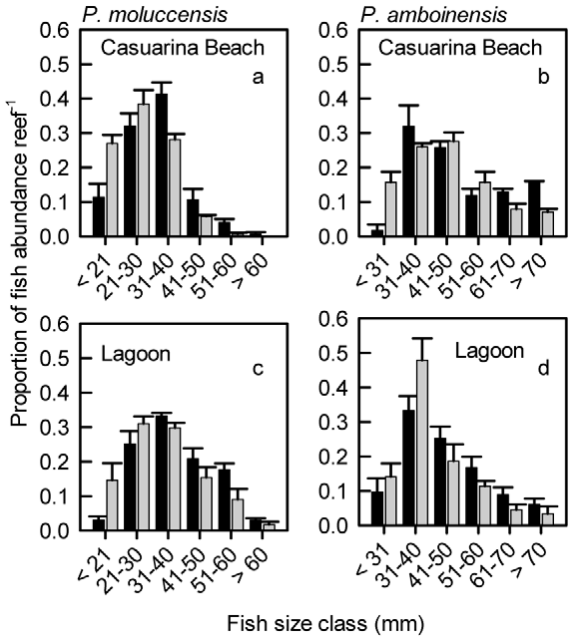
Mean (± s.e.) proportion of the number of damselfish per reef per fish size class for two species. Lemon damselfish *Pomacentrus moluccensis* are from the Lagoon (a) and Casuarina Beach (b) sites and ambon damselfish *P. amboinensis* are from the Lagoon (c) and Casuarina Beach (d) sites on reefs with cleaner wrasse *Labroides dimidiatus* present (dark grey bars) and absent (light grey bars). Data were analysed as number of fish per size class (total length) per reef but are presented here as proportions for ease of comparison between cleaner fish treatments. Number of reefs sampled according to *L. dimidiatus* presence. *P. moluccensis:* Lagoon *n* = 6 present, *n* = 5 absent; Casuarina Beach *n* = 3 present, *n* = 2 absent. *P. amboinensis*: Two reefs with small sample sizes were omitted (see results for details). Lagoon *n* = 6 present, *n* = 4 absent; Casuarina Beach *n* = 2 present, *n* = 2 absent.

Size frequency distributions of *P. moluccensis* per reef differed with cleaner presence (VGLM, see methods for definitions of statistical terms: χ^2^ = 35.4, df = 5, *P*<0.001), with the mean abundance per size class on reefs without cleaners skewed toward smaller individuals, compared with control reefs (Fig. 1a, c). *P. moluccensis* abundance did not differ between removal (208.0±29.4, least square mean ± s.e. per reef, here and hereafter) and control reefs (265.3±25.7) (*F_1,12_ = *2.3464, *P* = 0.1515); however, abundance per reef was higher at Casuarina Beach (292.79±34.0) compared with the Lagoon (180.5±22.6) (*P* = 0.0185, Table S1b).

The size distributions of *P. amboinensis* did not differ with cleaner presence (VGLM: χ^ 2^ = 10.0, df = 5, *P* = 0.075), possibly due to the small number of individuals on two reefs (removal reef 3, n = 7; control reef 16, n = 7). When these reefs were omitted, the size distributions of *P. amboinensis* were affected by cleaner presence (VGLM: χ^ 2^ = 11.7; df = 5; *P* = 0.039) (Fig. 1b, d), as for *P. moluccensis*. *P. amboinensis* abundance per reef did not differ between removal (52.1±11.9, least square mean ± s.e. per reef, here and hereafter) and control reefs (59.4±8.5) (*F_1,10_ = *0.2783, *P* = 0.6093) or between sites (*P* = 0.6783, Table S1d).

For both damselfishes, size distributions per reef differed between sites (*P. moluccensis*: χ^2^ = 54.6, df = 5, *P*<0.001; *P. amboinensis*: χ^ 2^ = 11.2, df = 5, *P* = 0.048), with smaller individuals at Casuarina Beach; the interaction between cleaner presence and site was not significant (*P. moluccensis*: χ^2^ = 1.7; df = 5; *P* = 0.8914; *P. amboinensis*: χ^ 2^ = 7.8, df = 5, *P* = 0.169).

### Fish abundance and species richness

In all analyses, no interactions between cleaner presence, site or time period of day (morning, noon, afternoon, see methods for exact times) were significant (*P>*0.05), except once where stated.

**Figure 2:**
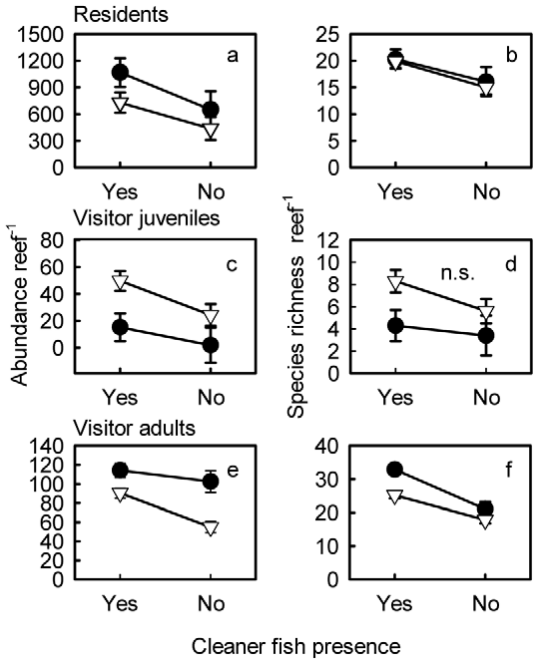
Least square mean (± s.e.) client fish numbers per reef. Fish are from reefs with and without cleaner fish *Labroides dimidiatus* present at Casuarina Beach (closed circles) and Lagoon (open triangles) sites. a) abundance of all residents, b) species richness of all residents. c) abundance of visitor juveniles, d) species richness of visitor juveniles, e) abundance of visitor adults, f) species richness of visitor adults. n.s.  =  cleaner fish presence not significant.

A total of thirty-eight resident species, mostly damselfishes (Pomacentridae, 32 species), were identified with 11 only found on reefs with cleaners (Table S2). After 18 months of manipulating cleaner presence, resident species richness per reef did not differ between removal (17.7±2.2, least square mean ± s.e. per reef, here and hereafter) and control reefs (19.0±2.0) (*F_1,11_ = *0.2167, *P* = 0.6507, Table S1f); whereas after 8.5 years, there were 23% fewer species per reef on removal (15.5±1.5) compared with control reefs (20.1±1.1) (*F_1,8_ = *5.9177, *P* = 0.0410, Table S1g; Fig. 2b). After 8.5 years, resident abundance per reef was 37% lower on removal (561.0±107.4) compared with control reefs (890.7±93.9) (*F_1,12_ = *5.8020, *P* = 0.0330, Table S1i; Fig. 2a). When resident species present on 15 or 16 reefs (*Ambliglyphidodon curacao*, *Neopomacentrus bankier, P. amboinensis, P. moluccensis*) were included as a random effect in the model, resident abundance per reef was lower on reefs without cleaners, compared to those with cleaners (GLM: *z = *−2.122, *P = *0.0338). Resident abundance also differed between sites (z* = *−3.844, *P = *0.0001), and increased with reef area (z* = *3.566, *P = *0.0004). Simpson's index of diversity per reef for residents did not differ between removal (0.78±0.03) and control reefs (0.77±0.02) (*F_1,8_ = *0.0602, *P* = 0.8124, Table S1j).

A total of nineteen species of juvenile visitor fishes were identified, with 7 and 1 found only on reefs with and without cleaners, respectively (Table S3). Juvenile visitor abundance per reef was 65% lower on removal (11.6±6.4, least square mean ± s.e. per reef, here and hereafter) compared with control reefs (33.2±5.6) (*F_1,11_ = *6.9072, *P* = 0.0235, Table S1l; Fig. 2c); however, species richness per reef did not differ between removal (4.9±1.0) and control reefs (6.7±0.8) (*F_1,11_ = *2.0736, *P* = 0.1777, Table S1n; Fig. 2d).

A total of 108 adult visitor fish species were identified, with 36 and 7 found only on reefs with and without cleaner fish, respectively (Table S4). Adult visitor abundance per reef was 23% lower on *L. dimidiatus* removal (78.8±6.3, least square mean ± s.e. per reef, here and hereafter) compared with control reefs (102.3±4.5) (*F_1,40_ = *10.0496, *P* = 0.0029, Table S1p; Fig. 2e) and species richness per reef was 33% lower on removal (19.4±1.2) compared with control reefs (29.0±0.9) (*F_1,32_ = *42.0191, *P*<0.0001, Fig. 2f) and increased with reef area (*P* = 0.0208, Table S1r). Total log_10_ (x+1) Acanthuridae abundance per reef was affected by cleaners (*F_1,42_ = *15.8983, *P* = 0.0003); when back-transformed, it was 66% lower on removal (7.5±0.2) compared with control reefs (22.1±0.2); it also increased with reef area (*P = *0.0044; Table S1t). When species present on 15 or 16 reefs (*Acanthurus* sp., *Cephalopholis cyanostigma, Coris aurilineata*, *Halichoeres melanurus, Stethojulis strigiventer, Thalassoma lunare*) were included as a random effect, visitor abundance per reef was lower on reefs without cleaners, compared to those with cleaners (GLM: *z = *−2.328, *P = *0.0199). Abundance also differed between sites (z* = *−2.681, *P = *0.0073), did not differ among times of day (*P>*0.05), and increased with reef area (z* = *1.826, *P = *0.0678). For the visitor Simpson's diversity index per reef, there was a significant interaction between cleaner presence and reef area (*F_1,30_ = *7.2425, *P* = 0.0115, Table S1v) due to a decreasing diversity with decreasing area on reefs without cleaners (slope = 0.00017, *t_19_ = 2.64*, *P = *0.0162) and no association with area on reefs with cleaners (slope = −0.0004, *t_18_ = −1.80*, *P = *0.0846). Similarly, after 18 months, visitor abundance was lower on removal (7.6±3.3) compared to control reefs (25.9±2.9) (*F_1,42_ = *19.0350, *P*<0.0001, Table S1x) and species richness was lower on removal (4.9±0.7) compared to control reefs (9.5±0.7) (*F_1,39_ = *24.0486, *P*<0.0001, Table S1z).

Total abundance per reef was higher at the Lagoon compared with the Casuarina Beach site for juvenile visitors (*P* = 0.0085, Table S1l; Fig. 2c); it was the opposite pattern for adult visitors (*P* =  <0.0001, Table S1p; Fig. 2e) and log10(x+1) Acanthuridae (*P = *0.0075, Table S1t). Species richness per reef was higher at Casuarina Beach for adult visitors (*P* = 0.0008, Table S1r; Fig. 2f). For adult visitors per reef, there was no effect of time period of day on abundance (*P* = 0.7382, Table S1p) or species richness (*P* = 0.8963, Table S1r).

## Discussion

Over an 8.5 year period, the removal from patch reefs of a single species - the cleaner wrasse *Labroides dimidiatus* - shifted the size distributions of two resident damselfish populations toward smaller individuals. This is the first demonstration that individual clients gain a fitness advantage from cleaners in the form of increased size. Cleaner absence also reduced the abundance and species richness of resident species and adult visitor species and the abundance of juvenile visitors. We argue these findings suggest that cleaner fish presence affects, directly or indirectly, the growth, survivorship, and/or recruitment of coral reef fishes; this is a first demonstration of potential mechanisms by which cleaners affect fish communities. To date, studies on the removal of key functional groups from coral reef fish communities have largely focused on the effects of large, mobile herbivores or predators due to their rapid worldwide depletion through human exploitation [22]–[24]. Here we demonstrate the dramatic impact of removing a single fish species that is small (maximum 8 cm total length) and not very abundant (1–5 adults per reef; mean±s.e. reef area: 131±34 m^2^, Table S5), but is nonetheless of great ecological importance. Cleaner fish remove ectoparasites from client fishes [1]; therefore, the profound influence that this species had on the local fish community indicates the powerful influence that ectoparasites have on coral reef fishes.

After 8.5 years, the size frequency distributions of the damselfishes *Pomacentrus moluccensis,* and of *P. amboinensis* when two reefs with very few individuals were omitted, were shifted towards smaller individuals on reefs without *L. dimidiatus*. In contrast, after the preliminary removal of *L. dimidiatus* there had been no difference in the estimated mean size of *P. moluccensis* per reef between treatments after 3 and 6 months. One likely consequence of this decreased size after 8.5 years is a decreased number and size of reproductively active adults per reef. Since female size and fecundity are highly correlated in damselfishes [25], reproductive output should be decreased on reefs without cleaner fish. *P. moluccensis* and other damselfishes are cleaned relatively infrequently compared with other clients included in the study [10], suggesting that any benefits of cleaning may be more pronounced in other, more frequently cleaned or heavily parasitised species.

We did not find an effect of cleaner fish presence on the abundance of *P. moluccensis* or *P. amboinensis.* This suggests that the smaller size of individuals in the absence of cleaners is not due to factors that increase abundance, such as increased post-settlement migration, a behaviour that is also rare in these damselfishes [26], nor to increased recruitment. It is possible that the populations of these species are constrained more by habitat and social dynamics as they live in corals [27] and in small social groups [28], respectively. Complex interactions between larval recruits and adults [29] and the large variation in recruitment events [26] may have also obscured any effect of cleaner fish presence. Multiple opposing indirect effects may also be acting concurrently; for example, the reduction in visitors (which included piscivores) on reefs without cleaner fish may increase prey survival.

Since the abundances of *P. moluccensis* or *P. amboinensis* were not affected by cleaner presence, the shifts in size distributions may have been due to decreased rates of growth where cleaner fish were absent. Indeed, in the absence of cleaners, *P. moluccensis* individuals had a lower growth rate and more parasitic copepod juveniles but this occurred only in larger individuals [30]. The risk of infection with other clients' ectoparasites may also be higher on reefs without cleaners if other clients are also more parasitized on such reefs [14], [19], [20]. The changes in size distributions of *P. moluccensis* and *P. amboinensis* are potentially also the consequence of indirect effects on fish health. For example, aggression from a piscivore towards nearby fish is reduced in the presence of *L. dimidiatus* [31]; this could, in turn, increase prey growth. This is the first time the presence of a cleaner organism has been shown to benefit (in terms of size) client individuals and confirms that cleaning is indeed a mutualism at this location, providing a firm foundation for studies of cooperation using this system (e.g. [2]).

While the species richness of resident fish (mostly damselfishes) per reef was not affected by cleaner presence after 18 months, it was lower after 8.5 years on reefs without cleaners. These results suggest that this effect of cleaners became apparent during this period. After 8.5 years, residents were also 37% less abundant, a parameter never previously measured. That the abundances of *P. moluccensis* and *P. amboinensis* were not affected by cleaner presence, however, indicates that only the abundance of some species was affected. Most resident species cannot and will not move readily to another part of the reef or patch reef to seek cleaning if no cleaning stations are available in their home range [32]. For these species, the benefits of being cleaned are perhaps not greater than the costs of traveling to a cleaner, which may include increased predation risk and energy output and loss of territory. On our isolated experimental reefs, swimming to another reef would involve a very high predation risk. Furthermore, the costs of not being cleaned may be lower for residents because they are smaller, with fewer and different ectoparasites compared to larger fishes [33]. Thus, the impact of cleaner fish removal may be less immediate in such fish and may only become detectable over a longer period. Consequently, changes to the community structure of resident fishes are likely not due to migration, but other factors, including reduced recruitment and mortality, associated with increased parasitism in the absence of cleaners but also unknown indirect effects. However, Bshary [4] found a reduction in the species richness of residents when *L. dimidiatus* was removed from reefs in the Red Sea, which was detected after 4 to 20 months. In Bshary's study, reefs were smaller (volume: 0.8 to 22 m^3^), the species composition of clients was different, and species richness was lower, factors which all may contribute to how quickly an effect of cleaning is observed. Furthermore, abundance was not measured in the Red Sea, so it is possible that individuals of absent species were replaced by individuals from the remaining species. Finally, Simpson's diversity index did not differ with cleaner presence for residents in our study; this suggests that the relative abundances across resident species were relatively even, regardless of cleaner presence.

This is the first study to consider juveniles separately from adult clients. The abundance of visitor juveniles was 65% lower in the absence of cleaners, suggesting that cleaner absence may decrease recruitment success or increase post-settlement migration of visitor species. Indeed, attacks by single parasitic gnathiid isopods decrease the successful settlement of *P. amboinensis* larvae (13mm, standard length), by affecting their performance as measured by swimming and oxygen consumption [12], and many visitor juveniles settle at a similar size (A.S.G. pers. obs.). If such gnathiids are not removed from fish by cleaner fish or gnathiid population densities are higher on reefs without cleaner fish, this could result in a reduction in juvenile abundance. *Dascyllus* damselfish larvae can recognise the cleaner fish *L. phthirophagus* [34]. Therefore, if larvae select reefs because of the presence of cleaner fish, cleaner absence may reduce their abundance. Over the long term, these effects on juveniles could lead to a reduction in the number recruited to the adult population. Differential survival and habitat choice during settlement are well known in damselfishes (e.g. [28]); however, the effect of cleaning remains unexplored.

For adults of visitor species, local abundance and species richness were lower on reefs without *L. dimidiatus* compared with control reefs both after 18 months and 8.5 years. This indicates the pattern likely persisted during this period. After 8.5 yrs, the Simpson's diversity index was also affected by cleaner presence but this was related to reef area, with a decrease with decreasing reef area on reefs without cleaners and no association with area on controls. Since visitor species richness increased with area, regardless of cleaner presence, while abundance did not, the lower species richness on smaller reefs may have made the diversity of such reefs more vulnerable to cleaner absence. This pattern may be related to habitat diversity, which often positively affects species richness [35]. The observed shifts in both abundance and richness may be non-independent results if richness increases with abundance simply based on random expectations of sampling.

Visitor clients, by definition, are more likely to modify their movements to search for cleaners, as these clients have the ability to select from several cleaning stations within their larger home ranges [4], [5]. For adults, particularly of larger species, the impact of cleaner fish removal may be more immediate as larger fish have higher ectoparasite loads [33]. Indeed, parasitic isopods on a caged visitor (*Hemigymnus melapterus*) at this location were higher in the absence of cleaners after 24 h and 12 d [19], [20]. Parasites are known to kill fish directly but might also do so indirectly by affecting metabolism [11], [36]–[38] or behaviour [13], [39]. Thus the decrease in visitor numbers could be due to parasite effects on survivorship. For both residents and adult visitors, when common species (see results for lists) were included as a random effect, there was still an effect of cleaners on abundance indicating the shift in species richness was not due to the loss of the more abundant fish species.

The effects of cleaner fish on clients are unlikely to have been a temporary effect due to disturbance from the removal of *L. dimidiatus* shortly before the observations were made as this occurred only on two reefs, and involved only two juvenile cleaner individuals. More importantly, collecting cleaners was done quickly and controls were similarly disturbed by counting *L. dimidiatus* on most surveys and leaving collecting equipment on the reef during counts.

The localised ecological effects of cleaner fish on fishes may have other indirect cascading effects on the reef community. Resident fishes consisted of herbivores and planktivores and visitors included herbivores, detritivores, piscivores, corallivores, and invertebrate predators. Herbivores were diverse and prominent on the reef (acanthurids, siganids, scarids, and some pomacentrids). The abundance of the most abundant and ubiquitous trophic group and family, the herbivorous Acanthuridae surgeonfishes (*Acanthurus, Ctenochaetus, Zebrasoma*), revealed abundance was 66% lower in the absence of cleaners. Herbivorous reef fishes limit the establishment and growth of algae that impede coral recruitment [40] and their removal has precipitated drastic shifts from coral to algal dominated systems [41], [42]. Visitor species also included piscivores (lethrinids, lutjanids, haemulids, holocentrids, serranids), invertebrate predators (e.g. labrids), and corallivores (e.g. chaetodonts) [8], [43]. Declines in the abundance of piscivores and invertebrate predators have been correlated with increases in fish prey abundance at fished sites [44] and have led to outbreaks of coral-eating starfish precipitating substantial declines in coral cover [45], respectively. Corallivores slow the progression of black-band disease [46]. Indirect effects on the benthic composition of the reefs are also likely to have further effects on the coral reef fish community, including the diverse benthos-associated fish community [8]. A more detailed study of fish foraging behavior and benthic composition is clearly required on these reefs.

The implications of this study are that (a) the behavioural interaction of cleaning of client fish by a relatively small number of small-sized fish has profound ecological consequences and (b) as ectoparasites are central to cleaning interactions, parasites can have a large effect on the population and community ecology of reef-fish. The presence of *L. dimidiatus* had remarkable effects on the local coral reef fish community that were considerably disproportionate to this species' small size and relatively low abundance. Potential mechanisms proposed for the above changes are effects on fish behaviour, movement, habitat choice, mortality, growth, and recruitment. Although this study measured only local effects, some effects may extend further. For example, the effect on the sizes of female fish, and hence the number of propagules produced [25], might increase dispersal to other areas. Furthermore, the effects of cleaner fish were consistent at two sites, suggesting that the strong effect of cleaner fish presence may also apply to abundance estimates of fish at a larger scale. The dramatic declines in fish and fish species numbers caused by the removal of this single cleaner fish species are comparable with significant fishing pressure [45], one of the leading known factors in coral reef community decline.

Positive interactions, including mutualisms, are considered important to communities because they make the environment, directly or indirectly, more favourable for associated species which in turn often facilitates the establishment of other species [3]. At larger regional scales, positive interactions enhance diversity via an increase in habitat diversity [47]. In our case, client fish provide cleaner fish with nutrients, as plant-mycorrhizal fungi, zooxanthellae-coral, and plant-pollinator associations do for their fungi, coral, and pollinator partners [47]. In exchange, in each case the other partner enjoys a more favourable environment. This may directly increase fish biodiversity, but there are likely also other indirect cascading benefits, for example habitat modification by various fishes [8], which may allow more species to coexist.

*L. dimidiatus* is one of the top ten most exported aquarium fish species to the United States of America and the United Kingdom [48]. The ecological effects of the large scale removal of *L. dimidiatus,* however, are unknown. Given the importance of the species *L. dimidiatus*, conservation and management strategies may need to also focus on the protection of this key species.

## Materials and Methods

### Ethics Statement

This study was carried out in strict accordance with the recommendations in the Guide for the Care and Use of Laboratory Animals of Australia. The protocol was approved by the Animal Experimentation Ethics Committee of the University of Queensland (Permit Number: SIB/821/08/URG). To ameliorate animal suffering, cleaner fish removed from experimental reefs were immediately placed in plastic bags with seawater and then released in a similar habitat.

### Removal of *L. dimidiatus*

We used 18 small spatially isolated patch reefs (3 to 7 m depth) located off Lizard Island (14°40′S, 145°28′E), GBR, Australia at two sites: in the southern lagoon (Lagoon, 12 reefs) and off the research station (Casuarina Beach, six reefs) [5]. Reef areas were calculated from satellite photographs using Optimax™ imaging software. Reefs were randomly allocated into nine removal (range 61–285 m^2^; mean ± s.e.: 131±34 m^2^) and nine control reefs (67–231 m^2^; 134±17 m^2^) in September 2000 [5]. All *L. dimidiatus* (1–5 adults, 0–3 juveniles per reef) were removed from removal reefs in September 2000. On 35 subsequent occasions at several month intervals, reefs were inspected for *L. dimidiatus* and any new recruits were removed. Subsequent removals occurred in 28% of all reef inspections, mostly in the summer, with 78% of removals involving 1–2 individuals reef^−1^, usually juveniles (Table S5). All reefs were surveyed for *L. dimidiatus* presence by swimming around the reef several times; on most occasions their abundance was also recorded on control reefs (Table S5). On removal reefs, cleaners were collected quickly with a barrier (1×1.5 m) and hand net, and placed in bags with seawater and released in similar habitat more 500 m from source reefs. On control reefs, sham removals were done by leaving collecting equipment on the reef while surveying it. In 2006, removal reefs 4 and 14 were dropped from the experiment after several observations of occasional visits of an adult *L. dimidiatus* from an adjacent reef and the repeated colonisation by an adult, respectively (A.S.G. pers. obs.). These reefs were the least isolated experimental reefs, being about 5 m from the nearest reef [15]. No such visits were ever observed on the other reefs during reef inspections and 100′s of hrs conducting other surveys (including video observations) [5]. Other cleaners, *Periclimines* or *Urocaridella* shrimp, were also observed on reef 11 (removal) and 15 (control), respectively, but not removed; their densities were low and so their presence unlikely to have a significant impact. All reefs were resurveyed 4–6, April 2009 for *L. dimidiatus.* A just-settled juvenile was observed on 4 April (reef 3), 4 d before the survey of visitors; it was so small it could not be captured with the available handnet and had disappeared the next day. Another juvenile was observed and collected 2 May (reef 8), after resident and visitor surveys and 2 d before damselfish size surveys.

### Fish surveys

Fish surveys alternated between randomly selected control and removal reefs within a site. Fishes were classified as either ‘visitors’ or ‘residents’ using lists adapted from Grutter *et al.* [5]. Visitors (adults only) (Table S4) were species presumed to move readily among reefs throughout the day, such as surgeonfishes (*Acanthurus* spp.), or species that are likely to spend weeks or months at one reef, but might move between reefs over a longer period, such as wrasses (Labridae), butterflyfishes (Chaetodontidae) and groupers (Serranidae). Because of their cryptic behaviour, cardinalfishes (Apogonidae), pipefishes (Sygnathidae), blennies (Blennidae), dottybacks (Pseudochromidae), and gobies (Gobidae) were not counted.

Adult visitors were identified and counted per reef (7–9 April 2009) in the morning (0900–1030 hrs), midday (1115–1245 hrs) and afternoon (1330–1500 hrs) following Grutter *et al.* [5]. An observer approached reefs slowly on snorkel, and circled the reef at a constant speed for 5–15 min, depending on reef size, during counts.

Resident fishes (Table S2), which are smaller, were counted once per reef (15–21 April 2009) (0930–430 hrs) by an observer on SCUBA for 60–120 min, depending on reef size. The observer systematically circled the reef counting one abundant species, or several less abundant species, in the same order, on each pass. Juveniles (total length <7 cm) of visitors were also counted at this time (Table S3).

### Size class distributions of *Pomacentrus moluccensis* and *P. amboinensis*

Damselfish *Pomacentrus moluccensis* and *P. amboinensis* were ideal resident client study species for the assessment of size class distributions because they occurred on all experimental reefs, their home ranges are small (<2 m in radius, Bruce Mapstone, unpubl. data), and migration is extremely limited [26].

All individuals per reef were grouped into 10 mm size classes. Size estimates were made by two observers (2–4 May 2009, 0830–1530 hrs). Prior to conducting estimates, sizes of 12–74 mm fish painted on metal disks placed on reefs were estimated. Errors in size estimates were examined following Bell *et al.* [49]; size class estimations were accurate in 96% of instances and inaccurate by one size class in 4%.

### Statistical analyses

Abundances of *P. moluccensis* and *P. amboinensis,* abundances and species richness of juvenile and adult visitors and residents, and the log_10_ (x+1) abundance of all (*Acanthurus, Ctenochaetus, Zebrasoma*) adult surgeonfishes (the most abundant and ubiquitous trophic group), per reef, were each analysed separately using analysis of covariance (ANCOVA) with two fixed factors (treatment: cleaners present or absent; site: Lagoon or Casuarina Beach), and reef area as a covariate; reef area was included as a covariate to test whether or not area significantly explained some of the variation in fish abundance or richness per reef. For adult visitors, time of day (morning, midday or afternoon) was a fixed factor; Acanthuridae abundance was log_10_(x+1) transformed to linearise data with area. Simpson's indices of diversity [50] were calculated for residents and adult visitors and analysed as above. To compare the results of this study to an earlier one conducted after 18 months of removing cleaner fish, visitor abundance and species richness and resident species richness (Table S1) and reef area from each study were analysed, separately. Prior to analyses, quantile-quantile plots of the residuals and plots of the residuals versus the fitted values were examined to check for normality and homogeneity of variances, respectively. All analyses began with a full model, with all possible interactions included; a final simplified model was selected by sequentially dropping highly non-significant interaction terms (*P*>0.25) following Quinn and Keough [51]. Factors were not removed from the final model, regardless of statistical significance; statistical results for the full and final models are presented (Table S1). These statistical analyses were done using JMP v.8.0 (2009) SAS Institute Inc.

Size classes of *P. moluccensis* and *P. amboinensis* were pooled, where necessary, to reduce the number of zero counts within any one size class to less than four reefs. A Dirichlet-Multinomial Vector Generalised Linear Modelling (VGLM) approach [52] was used as it allowed the analysis of the distribution of fish body sizes based on size-classed total abundance data with different numbers of fish at the different reefs [53]; separate analyses per species were used, with cleaner treatment and site as fixed factors and reef area as a covariate. To determine whether fish size varied with cleaner presence, an analysis of variance (ANOVA), with cleaner presence, site and duration of removal as fixed factors, and reef as a random factor was used.

To evaluate if fish abundance was affected by cleaners, with species taken into account, species that were present on 15 or 16 reefs were used. Separate generalised linear mixed models (GLMM) with a Poisson distribution were used for residents and adult visitors, with treatment, site, and time (visitors only) as fixed factors, species identity and reef as random variables, and reef area as a covariate. These statistical analyses were done using R v2.9.0 [54].

## Supporting Information

Table S1 Statistical results for fish abundance, species richness, and Simpson's diversity index analyses.
(DOC)

Table S2 Species list of site-attached resident fishes surveyed.
(DOC)

Table S3 Species list of juvenile visitor fishes surveyed.
(DOC)

Table S4 Species list of adult visitor fishes surveyed.
(DOC)

Table S5 Experimental reefs with number of cleaner fish present or removed.
(DOC)
