# Supplementary material for: Long-Term Effects of the Cleaner Fish Labroides dimidiatus on Coral Reef Fish Communities
Source: PLoS One. 2011 Jun 24;6(6):e21201. doi: 10.1371/journal.pone.0021201 (PMC3123342; doi:10.1371/journal.pone.0021201)
Supplement: Table S1 — Statistical results for fish abundance, species richness, and Simpson's diversity index analyses. (DOC) [file pone.0021201.s001.doc]

Table S1. Statistical results of multifactor analyses of covariance (ANCOVA). All factors are fixed and reef area is a covariate. *P* values in bold are ones presented in main text. * = *P <* 0.05. Cleaners = cleaner fish present or absent, Site = Casuarina Beach or Lagoon, Time = time of day: morning (0900-1030 hrs), midday (1115-1245 hrs) and afternoon (1330-1500 hrs).

1. *Pomacentrus moluccensis* abundance per reef. Full two factor ANCOVA model.

| Source | Numerator DF | Denominator DF | Sum of Squares | *F* | *P* |
| --- | --- | --- | --- | --- | --- |
| Cleaners | 1 | 8 | 6364.370 | 0.8871 | 0.3738 |
| Site | 1 | 8 | 30316.139 | 4.2254 | 0.0739 |
| Cleaners*Site | 1 | 8 | 368.237 | 0.0513 | 0.8265 |
| Area | 1 | 8 | 28080.029 | 3.9138 | 0.0833 |
| Cleaners*Area | 1 | 8 | 5220.062 | 0.7276 | 0.4185 |
| Site*Area | 1 | 8 | 669.592 | 0.0933 | 0.7678 |
| Cleaners*Site*Area | 1 | 8 | 3590.757 | 0.5005 | 0.4994 |

1. *Pomacentrus moluccensis* abundance per reef. Simplified two factor ANCOVA model.

| Source | Numerator DF | Denominator DF | Sum of Squares | *F* | *P* |
| --- | --- | --- | --- | --- | --- |
| Cleaners | 1 | 12 | 12911.993 | 2.3464 | **0.1515** |
| Site | 1 | 12 | 40818.893 | 7.4178 | **0.0185*** |
| Area | 1 | 12 | 65165.520 | 11.8423 | 0.0049* |

1. *Pomacentrus amboinensis* abundance per reef. Full two factor ANCOVA model.

| Source | Numerator DF | Denominator DF | Sum of Squares | *F* | *P* |
| --- | --- | --- | --- | --- | --- |
| Cleaners | 1 | 8 | 16.7588 | 0.0258 | 0.8763 |
| Site | 1 | 8 | 82.7281 | 0.1275 | 0.7303 |
| Cleaners*Site | 1 | 8 | 1477.9696 | 2.2778 | 0.1697 |
| Area | 1 | 8 | 346.6875 | 0.5343 | 0.4857 |
| Cleaners*Area | 1 | 8 | 181.4638 | 0.2797 | 0.6113 |
| Site*Area | 1 | 8 | 847.6118 | 1.3063 | 0.2861 |
| Cleaners*Site*Area | 1 | 8 | 385.1813 | 0.5936 | 0.4632 |

1. *Pomacentrus amboinensis* abundance per reef. Simplified two factor ANCOVA model.

| Source | Numerator DF | Denominator DF | Sum of Squares | *F* | *P* |
| --- | --- | --- | --- | --- | --- |
| Cleaners | 1 | 10 | 155.8899 | 0.2783 | **0.6093** |
| Site | 1 | 10 | 102.1953 | 0.1824 | **0.6783** |
| Cleaners*Site | 1 | 10 | 1236.6362 | 2.2074 | 0.1682 |
| Area | 1 | 10 | 963.2344 | 1.7194 | 0.2191 |
| Site*Area | 1 | 10 | 2087.3586 | 3.7260 | 0.0824 |

1. Resident diversity per reef in 2002 (using diversity and area from Grutter et al. 2003). Full two factor ANCOVA model.

| Source | Numerator DF | Denominator DF | Sum of Squares | *F* | *P* |
| --- | --- | --- | --- | --- | --- |
| Cleaners | 1 | 8 | 4.154966 | 0.1375 | 0.7204 |
| Site | 1 | 8 | 1.230152 | 0.0407 | 0.8451 |
| Cleaners*Site | 1 | 8 | 9.853870 | 0.3262 | 0.5836 |
| Area | 1 | 8 | 21.818588 | 0.7222 | 0.4201 |
| Cleaners*Area | 1 | 8 | 0.317182 | 0.0105 | 0.9209 |
| Site*Area | 1 | 8 | 4.672704 | 0.1547 | 0.7044 |
| Cleaners*Site*Area | 1 | 8 | 27.612281 | 0.9140 | 0.3671 |

1. Resident diversity per reef in 2002 (using diversity and area from Grutter et al. 2003). Simplified two factor ANCOVA model.

| Source | Numerator DF | Denominator DF | Sum of Squares | *F* | *P* |
| --- | --- | --- | --- | --- | --- |
| Cleaners | 1 | 11 | 5.909475 | 0.2167 | **0.6507** |
| Site | 1 | 11 | 3.735843 | 0.1370 | 0.7183 |
| Area | 1 | 11 | 11.490430 | 0.4213 | 0.5296 |
| Site*Area | 1 | 11 | 58.640955 | 2.1500 | 0.1706 |

1. Resident fish diversity per reef. Full and final two factor ANCOVA model.

| Source | Numerator DF | Denominator DF | Sum of Squares | *F* | *P* |
| --- | --- | --- | --- | --- | --- |
| Cleaners | 1 | 8 | 50.395702 | 5.9177 | **0.0410*** |
| Site | 1 | 8 | 1.197990 | 0.1407 | 0.7174 |
| Cleaners*Site | 1 | 8 | 0.286760 | 0.0337 | 0.8590 |
| Area | 1 | 8 | 1.831580 | 0.2151 | 0.6552 |
| Cleaners*Area | 1 | 8 | 11.145370 | 1.3087 | 0.2857 |
| Site*Area | 1 | 8 | 24.070001 | 2.8264 | 0.1312 |
| Cleaners*Site*Area | 1 | 8 | 17.848280 | 2.0958 | 0.1857 |

1. Resident fish abundance per reef. Full two factor ANCOVA.

| Source | Numerator DF | Denominator DF | Sum of Squares | *F* | *P* |
| --- | --- | --- | --- | --- | --- |
| Cleaners | 1 | 8 | 370045.26 | 3.7366 | 0.0893 |
| Site | 1 | 8 | 210605.97 | 2.1266 | 0.1829 |
| Cleaners*Site | 1 | 8 | 26757.58 | 0.2702 | 0.6173 |
| Area | 1 | 8 | 3288.85 | 0.0332 | 0.8599 |
| Cleaners*Area | 1 | 8 | 62244.53 | 0.6285 | 0.4508 |
| Site*Area | 1 | 8 | 50357.10 | 0.5085 | 0.4961 |
| Cleaners*Site*Area | 1 | 8 | 54319.48 | 0.5485 | 0.4801 |

1. Resident fish abundance per reef. Simplified two factor ANCOVA.

| Source | Numerator DF | Denominator DF | Sum of Squares | *F* | *P* |
| --- | --- | --- | --- | --- | --- |
| Cleaners | 1 | 12 | 426749.01 | 5.8020 | **0.0330*** |
| Site | 1 | 12 | 268321.19 | 3.6481 | 0.0803 |
| Area | 1 | 12 | 250426.05 | 3.4048 | 0.0898 |

1. Simpson index of diversity using abundance per resident fish species. Full and final two factor ANCOVA model.

| Source | Numerator DF | Denominator DF | Sum of Squares | *F* | *P* |
| --- | --- | --- | --- | --- | --- |
| Cleaners | 1 | 8 | 0.00027592 | 0.0602 | **0.8124** |
| Site | 1 | 8 | 0.00034921 | 0.0761 | 0.7896 |
| Cleaners*Site | 1 | 8 | 0.00058575 | 0.1277 | 0.7301 |
| Area | 1 | 8 | 0.00734071 | 1.6006 | 0.2414 |
| Cleaners*Area | 1 | 8 | 0.00812706 | 1.7720 | 0.2198 |
| Site*Area | 1 | 8 | 0.00999712 | 2.1798 | 0.1781 |
| Cleaners*Site*Area | 1 | 8 | 0.01743371 | 3.8013 | 0.0870 |

1. Juvenile visitor fish abundance per reef. Full two factor ANCOVA.

| Source | Numerator DF | Denominator DF | Sum of Squares | *F* | *P* |
| --- | --- | --- | --- | --- | --- |
| Cleaners | 1 | 8 | 677.8891 | 2.3288 | 0.1655 |
| Site | 1 | 8 | 1257.7292 | 4.3207 | 0.0713 |
| Cleaners*Site | 1 | 8 | 264.8061 | 0.9097 | 0.3681 |
| Area | 1 | 8 | 66.6684 | 0.2290 | 0.6451 |
| Cleaners*Area | 1 | 8 | 81.0000 | 0.2783 | 0.6122 |
| Site*Area | 1 | 8 | 279.1138 | 0.9588 | 0.3562 |
| Cleaners*Site*Area | 1 | 8 | 70.8230 | 0.2433 | 0.6351 |

1. Juvenile visitor fish abundance per reef. Simplified two factor ANCOVA.

| Source | Numerator DF | Denominator DF | Sum of Squares | *F* | *P* |
| --- | --- | --- | --- | --- | --- |
| Cleaners | 1 | 11 | 1818.6126 | 6.9072 | **0.0235*** |
| Site | 1 | 11 | 2690.2331 | 10.2176 | **0.0085*** |
| Area | 1 | 11 | 1728.9270 | 6.5665 | 0.0264* |
| Cleaners*Area | 1 | 11 | 717.7893 | 2.7262 | 0.1269 |

1. Juvenile visitor fish diversity per reef. Full two factor ANCOVA.

| Source | Numerator DF | Denominator DF | Sum of Squares | *F* | *P* |
| --- | --- | --- | --- | --- | --- |
| Cleaners | 1 | 8 | 5.236148 | 0.8942 | 0.3720 |
| Site | 1 | 8 | 10.373430 | 1.7715 | 0.2199 |
| Cleaners*Site | 1 | 8 | 3.746561 | 0.6398 | 0.4469 |
| Area | 1 | 8 | 9.211657 | 1.5731 | 0.2452 |
| Cleaners*Area | 1 | 8 | 1.578020 | 0.2695 | 0.6177 |
| Site*Area | 1 | 8 | 17.512801 | 2.9906 | 0.1220 |
| Cleaners*Site*Area | 1 | 8 | 4.213041 | 0.7195 | 0.4210 |

1. Juvenile visitor fish diversity per reef. Simplified two factor ANCOVA.

| Source | Numerator DF | Denominator DF | Sum of Squares | *F* | *P* |
| --- | --- | --- | --- | --- | --- |
| Cleaners | 1 | 11 | 11.179551 | 2.0736 | **0.1777** |
| Site | 1 | 11 | 18.334160 | 3.4007 | 0.0922 |
| Area | 1 | 11 | 3.151991 | 0.5846 | 0.4606 |
| Site*Area | 1 | 11 | 8.816188 | 1.6353 | 0.2273 |

1. Visitor fish abundance per reef. Full three factor ANCOVA.

| Source | Numerator DF | Denominator DF | Sum of Squares | *F* | *P* |
| --- | --- | --- | --- | --- | --- |
| Cleaners | 1 | 24 | 5379.1094 | 8.3112 | 0.0082* |
| Site | 1 | 24 | 8913.0910 | 13.7715 | 0.0011* |
| Cleaners*Site | 1 | 24 | 576.2152 | 0.8903 | 0.3548 |
| Time | 2 | 24 | 201.1325 | 0.1554 | 0.8569 |
| Cleaners*Time | 2 | 24 | 823.7548 | 0.6364 | 0.5379 |
| Site*Time | 2 | 24 | 1376.7541 | 1.0636 | 0.3609 |
| Cleaners*Site*Time | 2 | 24 | 254.6365 | 0.1967 | 0.8227 |
| Area | 1 | 24 | 135.1581 | 0.2088 | 0.6518 |
| Cleaners*Area | 1 | 24 | 273.2599 | 0.4222 | 0.5220 |
| Site*Area | 1 | 24 | 4911.2749 | 7.5884 | 0.0110* |
| Cleaners*Site*Area | 1 | 24 | 766.1946 | 1.1838 | 0.2874 |
| Time*Area | 2 | 24 | 337.7449 | 0.2609 | 0.7725 |
| Cleaners*Time*Area | 2 | 24 | 61.0024 | 0.0471 | 0.9541 |
| Site*Time*Area | 2 | 24 | 248.2897 | 0.1918 | 0.8267 |
| Cleaners*Site*Time*Area | 2 | 24 | 50.9125 | 0.0393 | 0.9615 |

1. Visitor fish abundance per reef. Simplified three factor ANCOVA.

| Source | Numerator DF | Denominator DF | Sum of Squares | *F* | *P* |
| --- | --- | --- | --- | --- | --- |
| Cleaners | 1 | 40 | 4734.6701 | 10.0496 | **0.0029*** |
| Site | 1 | 40 | 9179.4508 | 19.4839 | **<.0001*** |
| Cleaners*Site | 1 | 40 | 1213.4785 | 2.5757 | 0.1164 |
| Time | 2 | 40 | 288.1667 | 0.3058 | **0.7382** |
| Area | 1 | 40 | 3.0926 | 0.0066 | 0.9358 |
| Site*Area | 1 | 40 | 4384.8315 | 9.3070 | 0.0040* |

1. Visitor fish diversity per reef. Full three factor ANCOVA.

| Source | Numerator DF | Denominator DF | Sum of Squares | *F* | *P* |
| --- | --- | --- | --- | --- | --- |
| Cleaners | 1 | 24 | 660.35926 | 34.0252 | <.0001* |
| Site | 1 | 24 | 214.23057 | 11.0383 | 0.0029* |
| Cleaners*Site | 1 | 24 | 35.56877 | 1.8327 | 0.1884 |
| Time | 2 | 24 | 7.43071 | 0.1914 | 0.8270 |
| Cleaners*Time | 2 | 24 | 21.02836 | 0.5417 | 0.5887 |
| Site*Time | 2 | 24 | 4.36272 | 0.1124 | 0.8942 |
| Cleaners*Site*Time | 2 | 24 | 14.94074 | 0.3849 | 0.6846 |
| Area | 1 | 24 | 93.01349 | 4.7926 | 0.0385* |
| Cleaners*Area | 1 | 24 | 34.39610 | 1.7723 | 0.1956 |
| Site*Area | 1 | 24 | 12.47767 | 0.6429 | 0.4305 |
| Cleaners*Site*Area | 1 | 24 | 27.78461 | 1.4316 | 0.2432 |
| Time*Area | 2 | 24 | 21.24984 | 0.5475 | 0.5855 |
| Cleaners*Time*Area | 2 | 24 | 1.04406 | 0.0269 | 0.9735 |
| Site*Time*Area | 2 | 24 | 42.17572 | 1.0866 | 0.3534 |
| Cleaners*Site*Time*Area | 2 | 24 | 2.44301 | 0.0629 | 0.9392 |

1. Visitor fish diversity per reef. Simplified three factor ANCOVA.

| Source | Numerator DF | Denominator DF | Sum of Squares | *F* | *P* |
| --- | --- | --- | --- | --- | --- |
| Cleaners | 1 | 32 | 660.35926 | 42.0191 | **<.0001*** |
| Site | 1 | 32 | 214.23057 | 13.6316 | **0.0008*** |
| Cleaners*Site | 1 | 32 | 35.56877 | 2.2633 | 0.1423 |
| Time | 2 | 32 | 3.45341 | 0.1099 | **0.8963** |
| Site*Time | 2 | 32 | 10.76012 | 0.3423 | 0.7127 |
| Area | 1 | 32 | 93.01349 | 5.9185 | **0.0208*** |
| Cleaners*Area | 1 | 32 | 34.39610 | 2.1886 | 0.1488 |
| Site*Area | 1 | 32 | 12.47767 | 0.7940 | 0.3796 |
| Cleaners*Site*Area | 1 | 32 | 27.78461 | 1.7680 | 0.1930 |
| Time*Area | 2 | 32 | 34.04818 | 1.0833 | 0.3506 |
| Site*Time*Area | 2 | 32 | 52.67417 | 1.6758 | 0.2032 |

1. Acanthuridae (adult visitor) log_10_ (x+1) abundance per reef. Full three factor ANCOVA.

| Source | Numerator DF | Denominator DF | Sum of Squares | *F* | *P* |
| --- | --- | --- | --- | --- | --- |
| Cleaners | 1 | 24 | 0.9521350 | 4.7133 | 0.0400* |
| Site | 1 | 24 | 1.2316095 | 6.0968 | 0.0210* |
| Cleaners*Site | 1 | 24 | 0.0902891 | 0.4470 | 0.5102 |
| Time | 2 | 24 | 0.0842848 | 0.2086 | 0.8132 |
| Cleaners*Time | 2 | 24 | 0.1637777 | 0.4054 | 0.6712 |
| Site*Time | 2 | 24 | 0.5377329 | 1.3310 | 0.2830 |
| Cleaners*Site*Time | 2 | 24 | 0.2312416 | 0.5724 | 0.5717 |
| Area | 1 | 24 | 0.1526600 | 0.7557 | 0.3933 |
| Cleaners*Area | 1 | 24 | 0.0258703 | 0.1281 | 0.7236 |
| Site*Area | 1 | 24 | 0.0762330 | 0.3774 | 0.5448 |
| Cleaners*Site*Area | 1 | 24 | 0.0001148 | 0.0006 | 0.9812 |
| Time*Area | 2 | 24 | 0.0721348 | 0.1785 | 0.8376 |
| Cleaners*Time*Area | 2 | 24 | 0.0082713 | 0.0205 | 0.9798 |
| Site*Time*Area | 2 | 24 | 0.0294960 | 0.0730 | 0.9298 |
| Cleaners*Site*Time*Area | 2 | 24 | 0.0201964 | 0.0500 | 0.9513 |

1. Acanthuridae (adult visitor) log_10_ (x+1) abundance per reef. Simplified three factor ANCOVA.

| Source | Numerator DF | Denominator DF | Sum of Squares | *F* | *P* |
| --- | --- | --- | --- | --- | --- |
| Cleaners | 1 | 42 | 2.2116580 | 15.8983 | **0.0003*** |
| Site | 1 | 42 | 1.0998857 | 7.9064 | **0.0075*** |
| Time | 2 | 42 | 0.0675949 | 0.2430 | 0.7854 |
| Area | 1 | 42 | 1.2638127 | 9.0848 | **0.0044*** |

1. Simpson’s index of diversity using abundance per visitor species per reef. Full three factor ANCOVA model.

| Source | Numerator DF | Denominator DF | Sum of Squares | *F* | *P* |
| --- | --- | --- | --- | --- | --- |
| Cleaners | 1 | 24 | 0.00002209 | 0.0090 | 0.9252 |
| Site | 1 | 24 | 0.00194727 | 0.7926 | 0.3821 |
| Cleaners*Site | 1 | 24 | 0.00030691 | 0.1249 | 0.7268 |
| Time | 2 | 24 | 0.00330222 | 0.6721 | 0.5200 |
| Cleaners*Time | 2 | 24 | 0.00420911 | 0.8567 | 0.4372 |
| Site*Time | 2 | 24 | 0.00304513 | 0.6198 | 0.5465 |
| Cleaners*Site*Time | 2 | 24 | 0.00360849 | 0.7344 | 0.4903 |
| Area | 1 | 24 | 0.00275750 | 1.1225 | 0.2999 |
| Cleaners*Area | 1 | 24 | 0.01556969 | 6.3377 | 0.0189* |
| Site*Area | 1 | 24 | 0.00117341 | 0.4776 | 0.4961 |
| Cleaners*Site*Area | 1 | 24 | 0.00517170 | 2.1052 | 0.1597 |
| Time*Area | 2 | 24 | 0.00081143 | 0.1651 | 0.8487 |
| Cleaners*Time*Area | 2 | 24 | 0.00101673 | 0.2069 | 0.8145 |
| Site*Time*Area | 2 | 24 | 0.00021773 | 0.0443 | 0.9567 |
| Cleaners*Site*Time*Area | 2 | 24 | 0.00101323 | 0.2062 | 0.8151 |

1. Simpson’s index of diversity using abundance per visitor species per reef. Simplified three factor ANCOVA model.

| Source | Numerator DF | Denominator DF | Sum of Squares | *F* | *P* |  |
| --- | --- | --- | --- | --- | --- | --- |
| Cleaners | 1 | 30 | 0.00002209 | 0.0103 | 0.9199 |  |
| Site | 1 | 30 | 0.00194727 | 0.9058 | 0.3488 |  |
| Cleaners*Site | 1 | 30 | 0.00030691 | 0.1428 | 0.7082 |  |
| Time | 2 | 30 | 0.00406721 | 0.9460 | 0.3996 |  |
| Cleaners*Time | 2 | 30 | 0.00760667 | 1.7692 | 0.1878 |  |
| Site*Time | 2 | 30 | 0.00405890 | 0.9440 | 0.4003 |  |
| Cleaners*Site*Time | 2 | 30 | 0.00683051 | 1.5887 | 0.2209 |  |
| Area | 1 | 30 | 0.00275750 | 1.2827 | 0.2664 |  |
| Cleaners*Area | 1 | 30 | 0.01556969 | 7.2425 | **0.0115*** |  |
| Site*Area | 1 | 30 | 0.00117341 | 0.5458 | 0.4658 |  |
| Cleaners*Site*Area | 1 | 30 | 0.00517170 | 2.4057 | 0.1314 |  |
| Time*Area | 2 | 30 | 0.00100578 | 0.2339 | 0.7928 |  |

1. Visitor fish abundance per reef in 2002 (using diversity and area from Grutter et al. 2003). Full three factor ANCOVA.

| Source | Numerator DF | Denominator DF | Sum of Squares | *F* | *P* |
| --- | --- | --- | --- | --- | --- |
| Cleaners | 1 | 24 | 2521.6288 | 8.0862 | 0.0090* |
| Site | 1 | 24 | 198.1311 | 0.6354 | 0.4332 |
| Cleaners*Site | 1 | 24 | 22.9146 | 0.0735 | 0.7887 |
| Time | 2 | 24 | 79.1253 | 0.1269 | 0.8814 |
| Cleaners*Time | 2 | 24 | 28.5139 | 0.0457 | 0.9554 |
| Site*Time | 2 | 24 | 315.1495 | 0.5053 | 0.6096 |
| Cleaners*Site*Time | 2 | 24 | 161.6078 | 0.2591 | 0.7739 |
| Area | 1 | 24 | 33.8724 | 0.1086 | 0.7446 |
| Cleaners*Area | 1 | 24 | 13.6834 | 0.0439 | 0.8358 |
| Site*Area | 1 | 24 | 92.3021 | 0.2960 | 0.5914 |
| Cleaners*Site*Area | 1 | 24 | 21.0474 | 0.0675 | 0.7972 |
| Time*Area | 2 | 24 | 129.0849 | 0.2070 | 0.8145 |
| Cleaners*Time*Area | 2 | 24 | 95.9184 | 0.1538 | 0.8583 |
| Site*Time*Area | 2 | 24 | 110.9982 | 0.1780 | 0.8381 |
| Cleaners*Site*Time*Area | 2 | 24 | 139.3528 | 0.2234 | 0.8014 |

1. Visitor fish abundance per reef in 2002 (using diversity and area from Grutter et al. 2003). Simplified three factor ANCOVA.

| Source | Numerator DF | Denominator DF | Sum of Squares | *F* | *P* |
| --- | --- | --- | --- | --- | --- |
| Cleaners | 1 | 42 | 3941.7187 | 19.0350 | **<.0001*** |
| Site | 1 | 42 | 181.9316 | 0.8786 | 0.3540 |
| Time | 2 | 42 | 292.6250 | 0.7066 | 0.4991 |
| Area | 1 | 42 | 660.7213 | 3.1907 | 0.0813 |

1. Visitor fish species richness per reef in 2002 (using diversity and area from Grutter et al. 2003). Full three factor ANCOVA.

| Source | Numerator DF | Denominator DF | Sum of Squares | *F* | *P* |
| --- | --- | --- | --- | --- | --- |
| Cleaners | 1 | 24 | 74.228893 | 7.3497 | 0.0122* |
| Site | 1 | 24 | 33.728673 | 3.3396 | 0.0801 |
| Cleaners*Site | 1 | 24 | 1.465499 | 0.1451 | 0.7066 |
| Time | 2 | 24 | 20.498506 | 1.0148 | 0.3775 |
| Cleaners*Time | 2 | 24 | 44.731058 | 2.2145 | 0.1310 |
| Site*Time | 2 | 24 | 63.979980 | 3.1675 | 0.0601 |
| Cleaners*Site*Time | 2 | 24 | 31.949897 | 1.5817 | 0.2263 |
| Area | 1 | 24 | 19.814031 | 1.9619 | 0.1741 |
| Cleaners*Area | 1 | 24 | 2.882873 | 0.2854 | 0.5981 |
| Site*Area | 1 | 24 | 0.017231 | 0.0017 | 0.9674 |
| Cleaners*Site*Area | 1 | 24 | 11.663528 | 1.1549 | 0.2932 |
| Time*Area | 2 | 24 | 23.840244 | 1.1803 | 0.3244 |
| Cleaners*Time*Area | 2 | 24 | 17.916285 | 0.8870 | 0.4250 |
| Site*Time*Area | 2 | 24 | 22.712753 | 1.1244 | 0.3414 |
| Cleaners*Site*Time*Area | 2 | 24 | 51.235860 | 2.5365 | 0.1001 |

1. Visitor fish species richness per reef in 2002 (using diversity and area from Grutter et al. 2003). Simplified three factor ANCOVA.

| Source | Numerator DF | Denominator DF | Sum of Squares | *F* | *P* |
| --- | --- | --- | --- | --- | --- |
| Cleaners | 1 | 39 | 235.42282 | 24.0486 | **<.0001*** |
| Site | 1 | 39 | 111.24851 | 11.3641 | 0.0017* |
| Time | 2 | 39 | 5.47803 | 0.2798 | 0.7574 |
| Site*Time | 2 | 39 | 42.56136 | 2.1738 | 0.1273 |
| Area | 1 | 39 | 29.55377 | 3.0189 | 0.0902 |
| Cleaners*Area | 1 | 39 | 21.30387 | 2.1762 | 0.1482 |
