# Supplementary material for: Long-Term Effects of the Cleaner Fish Labroides dimidiatus on Coral Reef Fish Communities
Source: PLoS One. 2011 Jun 24;6(6):e21201. doi: 10.1371/journal.pone.0021201 (PMC3123342; doi:10.1371/journal.pone.0021201)
Supplement: Table S2 — Species list of site-attached resident fishes surveyed. (DOC) [file pone.0021201.s002.doc]

## Table S2 *All resident fishes observed on study reefs.*

| ***Fish Family*** | ***Fish species*** |
| --- | --- |
|
| Centriscidae | *Aeoliscus strigatus** |
| Chaetodontidae | *Heniochus acuminatus** |
| Labridae | *Stethojulis bandanensis** |
| Pomacentridae | *Acanthochromis polyacanthus* |
| Pomacentridae | *Amblyglyphidodon curacao* |
| Pomacentridae | *Amphiprion clarkii** |
| Pomacentridae | *Amphiprion clarkia* |
| Pomacentridae | *Chromis viridis* or *atripectoralis* |
| Pomacentridae | *Chrysiptera rollandi* |
| Pomacentridae | *Chrysiptera talboti** |
| Pomacentridae | *Dascyllus aruanus* |
| Pomacentridae | *Dascyllus reticulatus* |
| Pomacentridae | *Dischistodus melanotus** |
| Pomacentridae | *Dischistodus prosopotaenia* |
| Pomacentridae | *Dischistodus pseudochrysopoecilus** |
| Pomacentridae | *Hemiglyphidodon plagiometopon* |
| Pomacentridae | *Neoglyphidodon melas* |
| Pomacentridae | *Neoglyphidodon nigroris* |
| Pomacentridae | *Neopomacentrus bankieri* |
| Pomacentridae | *Neopomacentrus cyanomos* |
| Pomacentridae | *Plectroglyphidodon lacrymatus* |
| Pomacentridae | *Pomacentrus adelus* |
| Pomacentridae | *Pomacentrus amboinensis* |
| Pomacentridae | *Pomacentrus australis* |
| Pomacentridae | *Pomacentrus bankanensis* |
| Pomacentridae | *Pomacentrus coelestis** |
| Pomacentridae | *Pomacentrus grammorhynchus* |
| Pomacentridae | *Pomacentrus lepidogenys** |
| Pomacentridae | *Pomacentrus moluccensis* |
| Pomacentridae | *Pomacentrus nagasakiensis* |
| Pomacentridae | *Pomacentrus pavo* |
| Pomacentridae | *Pomacentrus simsiang* |
| Pomacentridae | *Premnas biaculeatus* |
| Pomacentridae | *Stegastes apicalis* |
| Pomacentridae | *Stegastes fasciolatus* |
| Ptereleotridae | *Ptereleotris evides** |
| Tetraodontidae | *Canthigaster papua** |
| Tetraodontidae | *Canthigaster valentini* |

* Species only recorded on reefs with *Labroides dimidiatus* present.
