# Supplementary material for: Long-Term Effects of the Cleaner Fish Labroides dimidiatus on Coral Reef Fish Communities
Source: PLoS One. 2011 Jun 24;6(6):e21201. doi: 10.1371/journal.pone.0021201 (PMC3123342; doi:10.1371/journal.pone.0021201)
Supplement: Table S3 — Species list of juvenile visitor fishes surveyed. (DOC) [file pone.0021201.s003.doc]

## Table S3 *All juvenile visitor fishes observed on study reefs.*

| ***Client Family*** | ***Client species*** |
| --- | --- |
| Acanthuridae | *Ctenochaetus binotatus* |
| Acanthuridae | *Ctenochaetus cyanocheilus** |
| Acanthuridae | *Zebrasoma veliferum* |
| Acanthuridae | Acanthuridae spp. |
| Balistidae | *Sufflamen chrysopterus** |
| Labridae | *Hemigymnus melapterus* |
| Labridae | *Labrichthys* spp. |
| Labridae | Labridae spp.*** |
| Lutjanidae | *Lutjanus carponotatus** |
| Lutjanidae | *Lutjanus fulviflamma* |
| Lutjanidae | *Lutjanus quinquelineatus* |
| Nemipteridae | *Scolopsis bilineatus* |
| Pomacanthidae | *Pomacanthus imperator** |
| Pomacanthidae | *Pomacanthus semicirculatus*† |
| Pomacanthidae | *Pomacanthus sexstriatus* |
| Scaridae | *Chlorurus sordidus* |
| Scaridae | Scaridae spp. |
| Siganidae | Siganidae spp.*** |
| Serranidae | *Plectropomus maculatus** |

* Species only recorded at reefs with *L. dimidiatus* present.

† Species only recorded at reefs without *L. dimidiatus* present.
