# Supplementary material for: Long-Term Effects of the Cleaner Fish Labroides dimidiatus on Coral Reef Fish Communities
Source: PLoS One. 2011 Jun 24;6(6):e21201. doi: 10.1371/journal.pone.0021201 (PMC3123342; doi:10.1371/journal.pone.0021201)
Supplement: Table S4 — Species list of adult visitor fishes surveyed. (DOC) [file pone.0021201.s004.doc]

## Table S4 *All adult visitor fishes observed on study reefs.*

| ***Client Family*** | ***Client species*** |
| --- | --- |
| Acanthuridae | *Acanthurus auranticavus* |
| Acanthuridae | *Acanthurus dussumieri* |
| Acanthuridae | *Acanthurus nigricauda* |
| Acanthuridae | *Acanthurus xanthopterus* |
| Acanthuridae | *Ctenochaetus striatus* |
| Acanthuridae | *Naso brevirostris** |
| Acanthuridae | *Naso tonganus** |
| Acanthuridae | *Naso unicornis* |
| Acanthuridae | *Zebrasoma veliferum* |
| Acanthuridae | *Acanthurus* sp.* |
| Balistidae | *Balistapus undulatus*† |
| Balistidae | *Balistoides viridescens** |
| Balistidae | *Melichthys vidua** |
| Balistidae | *Sufflamen fraenatus* |
| Chaetodontidae | *Chaetodon auriga* |
| Chaetodontidae | *Chaetodon baronessa** |
| Chaetodontidae | *Chaetodon ephippium** |
| Chaetodontidae | *Chaetodon lineolatus* |
| Chaetodontidae | *Chaetodon lunulatus* |
| Chaetodontidae | *Chaetodon ocellicaudus** |
| Chaetodontidae | *Chaetodon plebeius** |
| Chaetodontidae | *Chaetodon rainfordi* |
| Chaetodontidae | *Chaetodon ulietensis* |
| Chaetodontidae | *Chaetodon vagabundus* |
| Chaetodontidae | *Chaetodon* spp. |
| Chaetodontidae | *Chelmon rostratus* |
| Carcharhinidae | *Carcharhinus melanopterus** |
| Carcharhinidae | *Triaenodon obesus** |
| Dasyatididae | *Pastinachus sephen* |
| Dasyatididae | *Taeniura lymma* |
| Ephippidae | *Platax teira** |
| Haemulidae | *Diagramma pictum* |
| Haemulidae | *Plectorhinchus chaetodonoides* |
| Haemulidae | *Plectorhinchus chrysotaenia* |
| Haemulidae | *Plectorhinchus gibbosus* |
| Haemulidae | *Plectorhinchus unicolor*† |
| Holocentridae | *Neoniphon samara* |
| Holocentridae | *Sargocentron spiniferum* |
| Holocentridae | Holocentridae spp. |
| Labridae | *Cheilinus fasciatus* |
| Labridae | *Choerodon fasciatus** |
| Labridae | *Choerodon graphicus* |
| Labridae | *Coris aurilineata* |
| Labridae | *Coris batuensis* |
| Labridae | *Epibulus insidiator** |
| Labridae | *Gomphosus varius** |
| Labridae | *Halichoeres argus* |
| Labridae | *Halichoeres margaritaceus* |
| Labridae | *Halichoeres melanurus* |
| Labridae | *Halichoeres nigrescens*† |
| Labridae | *Halichoeres trimaculatus** |
| Labridae | *Hemigymnus melapterus* |
| Labridae | *Stethojulis strigiventer* |
| Labridae | *Thalassoma hardwicke** |
| Labridae | *Thalassoma lunare* |
| Labridae | *Thalassoma lutescens*† |
| Mullidae | *Mulloidichthys flavolineatus** |
| Mullidae | *Parupeneus barberinus* |
| Mullidae | *Parupeneus indicus* |
| Mullidae | *Parupeneus multifasciatus** |
| Mullidae | *Parupeneus pleurostigma* |
| Nemipteridae | *Monotaxis grandoculis*† |
| Nemipteridae | *Scolopsis bilineatus* |
| Nemipteridae | *Scolopsis temporalis* |
| Lethrinidae | *Gymnocranius grandoculis* |
| Lethrinidae | *Lethrinus harak* |
| Lethrinidae | *Lethrinus microdon* |
| Lethrinidae | *Lethrinus nebulosus* |
| Lethrinidae | *Lethrinus ornatus* |
| Lethrinidae | *Lethrinus* spp. |
| Lutjanidae | *Lutjanus bohar* |
| Lutjanidae | *Lutjanus carponotatus* |
| Lutjanidae | *Lutjanus fulviflamma* |
| Lutjanidae | *Lutjanus fulvus** |
| Lutjanidae | *Lutjanus quinquelineatus** |
| Lutjanidae | *Lutjanus russelli* |
| Lutjanidae | *Lutjanus vitta** |
| Lutjanidae | *Symphorus nematophorus*† |
| Lutjanidae | *Lutjanus* spp. |
| Pomacanthidae | *Pomacanthus sexstriatus* |
| Pomacanthidae | Pomacanthidae spp.* |
| Scaridae | *Chlorurus bleekeri** |
| Scaridae | *Chlorurus sordidus** |
| Scaridae | *Hipposcarus longiceps** |
| Scaridae | *Scarus altipinnus** |
| Scaridae | *Scarus chameleon* |
| Scaridae | *Scarus flavipecoralis* |
| Scaridae | *Scarus frenatus** |
| Scaridae | *Scarus ghobban* |
| Scaridae | *Scarus niger** |
| Scaridae | *Scarus rivulatus* |
| Scaridae | *Scarus schlegeli** |
| Scaridae | Scaridae spp. |
| Siganidae | *Siganus argenteus** |
| Siganidae | *Siganus corallinus** |
| Siganidae | *Siganus doliatus* |
| Siganidae | *Siganus lineatus* |
| Siganidae | *Siganus puellus* |
| Siganidae | *Siganus punctatissimus* |
| Siganidae | *Siganus vulpinus** |
| Serranidae | *Cephalopholis cyanostigma* |
| Serranidae | *Cephalopholis microprion** |
| Serranidae | *Epinephelus cyanopodus** |
| Serranidae | *Epinephelus tukula** |
| Serranidae | *Plectropomus leopardus* |
| Serranidae | *Plectropomus maculatus* |
| Tetraodontidae | *Arothron stellatus*† |
| Zanclidae | *Zanclus cornutus* |

* Species only recorded at reefs with *L. dimidiatus* present.

† Species only recorded at reefs without *L. dimidiatus* present.
