# Supplementary material for: Long-Term Effects of the Cleaner Fish Labroides dimidiatus on Coral Reef Fish Communities
Source: PLoS One. 2011 Jun 24;6(6):e21201. doi: 10.1371/journal.pone.0021201 (PMC3123342; doi:10.1371/journal.pone.0021201)
Supplement: Table S5 — Experimental reefs with number of cleaner fish present or removed. (DOC) [file pone.0021201.s005.doc]

Table S5 Surveys of *Labroides dimidiatus* (adults/juveniles), September 2000 to April 2009.

| **Reef** | **1** | **5** | **9** | **10** | **12** | **13** | **7** | **15** | **16** | **2** | **3** | **11** | **17** | **18** | **6** | **8** |  |
| --- | --- | --- | --- | --- | --- | --- | --- | --- | --- | --- | --- | --- | --- | --- | --- | --- | --- |
| **Treatment** | C | C | C | C | C | C | C | C | C | R | R | R | R | R | R | R |  |
| **Location** | L | L | L | L | L | L | CB | CB | CB | L | L | L | L | L | CB | CB |  |
| **Area (m^2^)** | 82 | 171 | 67 | 131 | 231 | 97 | 111 | 164 | 149 | 85 | 64 | 61 | 285 | 65 | 127 | 227 |  |
| **Sept. 2000** | 1/1 | 3/0 | 1/0 | 2/1 | 1/1 | 3/1 | 5/1 | 2/1 | 5/1 | **-3/0** | -**1/1** | **-2/1** | **-4/3** | **-2/1** | **-3/1** | **-5/0** |  |
| **Nov. 2000** | Na | Na | Na | Na | Na | Na | Na | Na | Na | 0/0 | 0/0 | 0/0 | 0/0 | 0/0 | 0/0 | 0/0 |  |
| **Jan. 2001** | 1/2 | 2/2 | 1/0 | 2/2 | 1/1 | 3/1 | 5/2 | 3/5 | 2/2 | 0/0 | 0/0 | 0/0 | 0/0 | 0/0 | 0/0 | 0/0 |  |
| **Apr. 2001** | Na | Na | Na | Na | Na | Na | Na | Na | Na | 0/0 | 0/0 | 0/0 | **-0/2** | 0/0 | 0/0 | 0/0 |  |
| **Aug. 2001** | 2/0 | 2/2 | 0/1 | 2/2 | 2/0 | 2/2 | 2/1 | 2/2 | 2/0 | 0/0 | 0/0 | 0/0 | 0/0 | 0//0 | 0/0 | 0/0 |  |
| **Nov. 2001** | 3/1 | 3/0 | 2/2 | 3/1 | 2/2 | 3/0 | 2/3 | 2/3 | 2/3 | 0/0 | **-0/2** | 0/0 | **-0/8** | **-0/5** | **-0/3** | **-0/4** |  |
| **Jan. 2002** | 2/1 | 3/0 | ½ | 2/2 | 2/2 | 2/1 | 0/8 | 2/4 | 2/3 | 0/0 | **-0/1** | 0/0 | **-0/1** | **-0/1** | 0/0 | **-0/3** |  |
| **Mar. 2002** | 2/1 | 2/1 | 2/1 | 2/1 | 2/2 | 2/0 | 3/3 | 3/4 | 3/2 | **-1/0** | 0/0 | 0/0 | **-0/1** | 0/0 | 0/0 | 0/0 |  |
| **Jun. 2002** | 2/0 | 2/0 | 3/1 | 4/0 | 3/1 | 3/0 | 2/0 | 5/1 | 3/0 | 0/0 | 0/0 | 0/0 | 0/0 | 0/0 | 0/0 | 0/0 |  |
| **Oct. 2002** | 1/1 | 2/0 | 2/0 | 3/0 | 4/0 | 3/0 | 3/1 | 4/1 | 4/2 | 0/0 | 0/0 | **-0/1** | 0/0 | **-0/1** | **-0/1** | **-0/1** |  |
| **Dec. 2002** | 2/0 | 1/0 | 2/1 | 2/1 | 3/1 | 2/1 | 4/0 | 4/1 | 4/1 | 0/0 | 0/0 | 0/0 | 0/0 | 0/0 | 0/0 | **-0/1** |  |
| **Jan. 2003** | 2/0 | 1/0 | 2/0 | 2/1 | 4/1 | 2/2 | 3/1 | 5/1 | 4/1 | 0/0 | 0/0 | 0/0 | **-0/2** | **-0/1** | 0/0 | 0/0 |  |
| **April 2003** | 1/0 | 1/0 | 2/0 | 2/0 | 3/0 | 2/0 | 3/1 | 3/2 | 1/0 | 0/0 | 0/0 | 0/0 | 0/0 | 0/0 | 0/0 | 0/0 |  |
| **Aug. 2003** | 3/1 | 0/0 | 2/0 | 2/0 | 4/1 | 2/0 | 3/1 | 3/1 | 4/0 | 0/0 | **-0/1** | 0/0 | **-0/1** | 0/0 | 0/0 | 0/0 |  |
| **Jan. 2004** | 2/1 | 1/0 | 2/0 | 3/0 | 3/2 | 1/0 | 5/1 | 3/0 | 5/1 | 0/0 | 0/0 | 0/0 | **-0/1** | 0/0 | 0/0 | 0/0 |  |
| **April 2004** | Na | Na | Na | Na | Na | Na | Na | Na | Na | Na | Na | Na | Na | Na | Na | Na |  |
| **Aug. 2004** | Na | Na | Na | Na | Na | Na | Na | Na | Na | 0/0 | 0/0 | 0/0 | 0/0 | 0/0 | 0/0 | 0/0 |  |
| **Nov. 2004** | 3/0 | 1/0 | 2/0 | 0/0 | 2/1 | 1/0 | 3/0 | 4/0 | 3/0 | 0/0 | **-1/1** | 0/0 | 0/0 | **-0/1** | **-0/1** | **-0/2** |  |
| **Jan. 2005** | 2/0 | 0/2 | 0/2 | 0/3 | 3/0 | 1/2 | Na | Na | Na | **-0/3** | **-0/3** | 0/0 | **-0/3** | 0/0 | **-0/2** | **-0/2** |  |
| **April 2005** | 2/0 | 1/0 | 1/0 | 2/0 | 2/2 | 2/0 | Na | Na | Na | 0/0 | **0/1** | 0/0 | **-3/0** | 0/0 | **-0/2** | 0/0 |  |
| **July 2005** | Na | Na | Na | Na | Na | Na | Na | Na | Na | 0/0 | **-0/1** | 0/0 | 0/0 | 0/0 | 0/0 | **-1/0** |  |
| **Nov. 2005** | Na | Na | Na | Na | Na | Na | Na | Na | Na | 0/0 | 0/0 | 0/0 | 0/0 | 0/0 | **-1/1** | **-0/1** |  |
| **Jan. 2006** | Na | Na | Na | Na | Na | Na | Na | Na | Na | 0/0 | **-0/2** | 0/0 | **-0/3** | 0/0 | **-0/1** | **-0/1** |  |
| **May 2006** | Na | Na | Na | Na | Na | Na | Na | Na | Na | 0/0 | **-0/1** | 0/0 | 0/0 | **-0/1** | 0/0 | **-0/1** |  |
| **Aug. 2006** | Na | Na | Na | Na | Na | Na | Na | Na | Na | 0/0 | 0/0 | 0/0 | 0/0 | 0/0 | 0/0 | **-0/1** |  |
| **Nov. 2006** | Na | Na | Na | Na | Na | Na | Na | Na | Na | 0/0 | 0/0 | 0/0 | 0/0 | 0/0 | 0/0 | 0/0 |  |
| **Dec. 2006** | Na | Na | Na | Na | Na | Na | Na | Na | Na | **-2/0** | 0/0 | 0/0 | 0/0 | 0/0 | 0/0 | 0/0 |  |
| **Jan. 2007** | Na | Na | Na | Na | Na | Na | Na | Na | Na | 0/0 | 0/0 | 0/0 | 0/0 | 0/0 | **-2/0** | 0/0 |  |
| **Apr. 2007** | 1/0 | Na | 2/1 | Na | 0/0 | 3/1 | 2/1 | 1/0 | 2/0 | **-1/0** | 0/0 | 0/0 | **-1/0** | 0/0 | 0/0 | 0/0 |  |
| **July 2007** | Na | Na | Na | Na | Na | Na | Na | Na | Na | 0/0 | **-0/1** | 0/0 | **-0/1** | **-0/1** | 0/0 | 0/0 |  |
| **Nov. 2007** | 1/0 | 1/0 | 0/2 | 2/0 | 0/2 | 4/0 | 0/5 | 0/3 | 0/3 | 0/0 | 0/0 | 0/0 | 0/0 | 0/0 | 0/0 | 0/0 |  |
| **Mar. 2008** | 2/0 | Na | 2/0 | 3/0 | 0/0 | 3/0 | Na | Na | 2/0 | **-0/1** | 0/0 | 0/0 | 0/0 | 0/0 | 0/0 | 0/0 |  |
| **July 2008** | 1/0 | 1/0 | 2/0 | 3/0 | 4/0 | 4/0 | 2/0 | 2/0 | 1/0 | 0/0 | 0/0 | 0/0 | 0/0 | **-2/0** | **-1/0** | 0/0 |  |
| **Oct. 2008** | 1/0 | 1/0 | 3/0 | 3/0 | 3/0 | 2/0 | 2/1 | 2/0 | 2/0 | **-1/2** | **-0/4** | 0/0 | 0/0 | **-0/1** | 0/0 | **-0/1** |  |
| **Jan. 2009** | 1/0 | 2/2 | 2/1 | 2/1 | 4/2 | 1/0 | 4/2 | 5/1 | 2/2 | 0/0 | **-0/1** | **-1/2** | **-3/1** | 0/0 | **-2/0** | **-1/1** |  |
| **Apr. 2009** | 1/0 | 3/0 | 2/2 | 2/1 | 2/1 | 2/1 | 4/2 | 3/0 | 4/0 | 0/0 | **-0/1** | 0/0 | 0/0 | 0/0 | 0/0 | **-0/1** |  |

C=Control, R=Removal. L= Lagoon, CB= Casuarina Beach. (-) and bold numbers indicate *L. dimidiatus* removed. Adults were 50 to 80 mm in total length (TL). Na=Data not available
